# Design and Characterization of the Surface Porous UHMWPE Composite Reinforced by Graphene Oxide

**DOI:** 10.3390/polym13040482

**Published:** 2021-02-03

**Authors:** Xiaohong Chen, Sheng Zhang, Lin Zhang, Ping Zhu, Gangqiang Zhang

**Affiliations:** 1 Institute of Advanced Materials, North China Electric Power University, Beijing 102206, China; 2 Ningbo Research Institute, Zhejiang University, Ningbo 315100, China; szhang1984@nit.zju.edu.cn; 3 College of Textile and Clothing, Institute of Functional Textiles and Advanced Materials, State Key Laboratory of Bio-Fibers and Eco-Textiles, Collaborative Innovation Center of Marine Biomass Fibers Materials and Textiles of Shandong Province, Qingdao University, Qingdao 266071, China; lzhang69@ncsu.edu (L.Z.); pzhu99@163.com (P.Z.); 4 Wilson College of Textiles, North Carolina State University, Raleigh, NC 27606, USA

**Keywords:** surface porous, GO nanosheets, NaCl pore-forming filler, tribology properties, UHMWPE composites

## Abstract

The surface porous ultrahigh molecular weight polyethylene (UHMWPE) composites were successfully fabricated with NaCl and graphene oxide (GO) in the hot-pressing procedure. The GO sheets were evenly dispersed in UHMWPE with the sedimentation method of GO in saturated NaCl. The morphologies, chemical compositions, mechanical, and tribological properties of GO and surface porous GO/NaCl/UHMWPE were investigated. The results show that GO sheet and NaCl could be evenly dispersed in UHMWPE. The regular pores are present on the surface of UHMWPE after NaCl dissolution in distilled water. The wear resistance properties are improved significantly, and the friction properties increased slightly with the addition of GO and NaCl.

## 1. Introduction

Artificial joints are used to replace the malfunctioning bones, enabling thousands of people to enjoy an active lifestyle [1]. The artificial joints are composed of two parts: metal and polymer. Ultra-high molecular weight polyethylene (UHMWPE) is the most common material used for the artificial joint bearing component, with the advantage of unique characteristics and favorable properties, such as biocompatibility, chemical stability, high wear resistance, and low friction, [2,3]. However, the low tribological properties of UHMWPE activate osteolysis and aseptic loosening, which lead to artificial joint replacement failure [4].

The polymer tribological properties can be improved by introducing filler materials to the polymer structure, resulting in mechanics modification [5,6]. Articular cartilage with porous structure has the advantage to provide human joints with excellent bearing capacity, bio-friction, and wear resistance performance [7]. Porous UHMWPE simulating as closely as possible the porous articular cartilage is a promising composite polymer used for various implant materials [8,9]. The various type and distribution of the filler materials are associated with the versatility of composite polymers. Maksimkin et al. [10] demonstrated that the porous structure of UHMWPE implants formed with the optimal method of monolithization at high pressure was a positive direction for implant in the case of filler formation and removal. Zhang et al. [11] fabricated UHMWPE microporous materials by loose sintering method and calculated the pore size by the face-centered cubic structure model. The results have shown that the average pore size and porosity increased with the increase of UHMWPE particle diameter, while the compressive strength and bulk density decreased.

Sodium chloride (NaCl) is one biological compatible and innocuous pore-forming filler to fabricate the porous structure formation in UHMWPE with thermal compression [12]. Pal et al. [13] prepared porous UHMWPE by mixing NaCl and hydroxyapatite powder as a pore-forming agent with hot isostatic pressing (HIP) and dissolving. Plumlee et al. [14] fabricated the porous UHMWPE with dry mixing of NaCl particles and UHMWPE powder by the hot pressing method after the leaching of NaCl. Zalepugina and Maksimkin et al. [15,16] obtained the porous multilayer UHMWPE with size controllable in subcritical water, which could be used as a medical device for reconstructive medicine. However, the research revealed that the porous formation in UHMWPE was associated with structure destruction, under low mechanical stresses [14]. After NaCl dissolution, the tribological properties of porous UHMWPE were similar to the porous articular cartilage. Unfortunately, due to the appearance of holes, the integrity of polyethylene is destroyed, which affects its mechanical properties. Hence, it is necessary to improve the mechanical properties of porous polyethylene. Carbon materials with excellent mechanical properties and out-performance lubrication characteristics could be used as the reinforcing filler for polymer [4,17,18,19,20]. Graphene oxide (GO) with a stacked two-dimensional structure became the research focus in the tribological field. Tai et al. [21] fabricated UHMWPE composited with GO to enhance the mechanical properties. The hardness and wear resistance of the GO/UHMWPE composite were improved significantly when the GO content is up to 1.0 wt.%. Suñer et al. [22] reported that the mechanical and wettability properties of GO/UHMWPE composite were enhanced compared to virgin UHMWPE. When the GO content is up to 0.5 wt.%, the composite exhibited higher characteristics. They [23] also assessed the biocompatibility of GO/UHMWPE wear particles. They found that the GO with higher concentrations (2 wt.%) led to a significant reduction in wear and GO to UHMWPE matrix did not significantly affect the inflammatory properties. Bahrami et al. [24] found that GO nanocomposite is a suitable filler and could be appropriately used in implant and artificial body due to the low coefficient of friction and wear rate of UHMWPE with the addition of GO. The rubbing surface morphological showed that GO produced an exfoliated structure without any agglomeration in the polymeric matrix. However, the dry GO sheets are different from evenly dispersed in UHMWPE powders. Suñer et al. [22] found suboptimal dispersion of the GO in the UHMWPE matrix when the GO content is above 0.5 wt.%. Therefore, it is an efficient approach to fabricate porous polyethylene that GO with the excellent reinforcement strength properties was utilized to compensate the damage of UHMWPE mechanical property due to the porous property. However, GO is hydrophilic with the water-soluble units, and it is difficult to evenly disperse in hydrophobic PE during the fabrication process. We found that GO dispersion could be mixed with NaCl solution and GO sheets with saturated NaCl could be aggregated in ethanol. It would be benefited for the GO dispersion with NaCl in PE.

The main purpose of this study is to fabricate PE materials with excellent friction and mechanical properties, NaCl is used as pore forming agent to endow PE material with a porous structure, and GO is used as the reinforcing agent to improve the PE mechanical properties. The NaCl and GO fillers were used to composite the UHMWPE to fabricate the surface porous GO/NaCl/UHMWPE composites with the sedimentation method of GO in saturated NaCl through the ethanol-assisted mixing and hot forming process. The mechanical and tribological properties of the UHMWPE and surface porous GO/NaCl/UHMWPE composites were evaluated. The effect of the GO on the tribological performance of the surface porous GO/UHMWPE was analyzed.

## 2. Materials and Experiments

### 2.1. Materials

UHMWPE powder and graphite powder (500 mesh, 99.85%) were obtained from Sinopharm Chemical Reagent Co. Ltd., Shanghai, China. The chemicals (H_2_SO_4_ 98%, H_3_PO_4_ 85%, KMnO_4_ 99%, HCl 37%, H_2_O_2_ 30%) used to synthesis GO were purchased from Sigma-Aldrich Co. LLC. The sodium chloride (NaCl) and ethanol solution were purchased from Sigma Aldrich Co. LLC, Shanghai, China. These chemicals were used without further treatment.

### 2.2. Synthesis of GO

The GO dispersions were synthesized following the Hummers method [25]. Graphite powder (1.5 g) was mixed with H_2_SO4 (98%,) and HCl in the ratio of 9:1 (180 mL and 20 mL). KMnO4 (10 g) was slowly added to this suspension and stirred at 50 °C with magnetic stirring evenly at 600 rpm. After 12 h, H_2_O_2_ (6 mL, 30%) and concentrated HCl (20 mL) were slowly dripped into this suspension. The mixture was settled for 12 h. The supernatant was moved out. Finally, the remaining mud was washed with distilled water until the pH value was 7. GO dispersions of a certain volume were dried in a vacuum oven at 45 °C. The drying GO powder was used for characterization.

### 2.3. Fabrication of GO/NaCl/UHMWPE Composites

**Figure 1:**
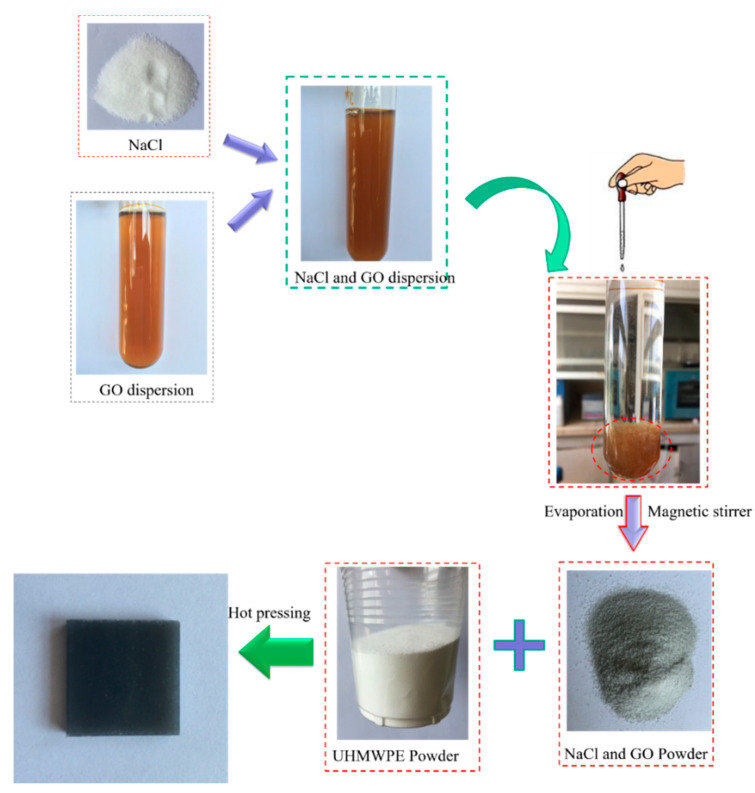
Schematic representation of the fabrication of surface porous graphene oxide (GO)/NaCl/ ultrahigh molecular weight polyethylene (UHMWPE) composites.

The schematic representation of surface porous GO/NaCl/UHMWPE composites fabrication is shown in Figure 1. The surface porous GO/NaCl/UHMWPE composites fabrication included the following steps: The cleaned GO dispersion was mixed with a saturated NaCl solution and stirred for 30 min. Then, the homogeneous NaCl and GO mixture was slowly dropped into ethanol solution. The GO and NaCl flocculate were deposited at the bottom of the test tube. Also, the flocculate deposition was dried via the rotary evaporation method at 70 °C to remove the ethanol. Furthermore, the supernatant was moved away, and the NaCl and GO dispersion was dried and ground into a powder. The dried GO/NaCl/UHMWPE powder and UHMWPE powder were fully mixed and treated by hot pressing to form a rectangular laminate. The compression was pre-treated at 7.5 MPa for 2 min and then conducted at 200 °C and 10 MPa for 2 h to form the GO /NaCl/UHMWPE laminate. Finally, the laminate was kept at room temperature for cooling and washed with distilled water and ethanol in the ultrasonic bath. A similar method was used to fabricate the virgin UHMWPE specimen. The mass ratios of specimens are shown in Table 1.

### 2.4. Characterization of GO and Surface Porous GO/NaCl/UHMWPE Composites

The size and thickness of GO sheets were measured by a multimode atomic force microscope (AFM) (nano-scope III, Bruker, Karlsruhe, Germany). The chemical compositions of GO and surface porous GO/UHMWPE were characterized by Fourier transform infrared spectroscopy (FT-IR) (Paragon 1000, Perkin Elmer, USA). The structure of GO and morphology of surface porous GO/NaCl/UHMWPE were observed by scanning electron microscope (SEM) (FEI Company, Hillsboro, Oregon, USA). The microstructure of GO and surface porous GO/NaCl/UHMWPE was performed by X-ray diffractometer (XRD) (2200/PC, Rigaku Corporation, Tokyo, Japan) under the condition of 2*θ* = 5–60°, λ = 0.154 nm. The microhardness experiments were carried out with the fabricated specimens using the Vicker microhardness tester. The load was 500 gf, and the holding time was 10 s. The water static contact angles of sample surfaces were measured according to the ISO 15989 standard. Five times tests were prepared at different locations.

### 2.5. Tribological Measurement

**Figure 2:**
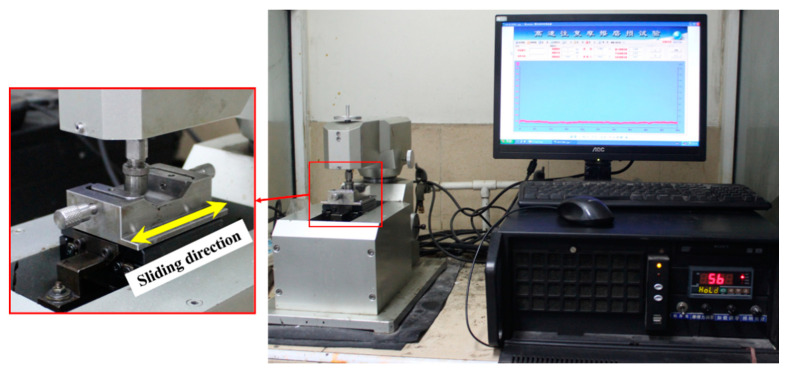
Digital photos of the reciprocating tribometer.

The tribological performance of the UHMWPE and surface porous GO/NaCl/UHMWPE composite were evaluated by the reciprocating tribometer (Lanzhou Institute of Chemical Physics, Lanzhou, China) as shown in Figure 2. The coefficient of friction is a significant factor in understanding the friction performance of the polymer. The elastic model of UHMWPE is less than that of the metal. Wear ratio is one important factor to evaluate the wear performance of the polymer. The friction force and the corresponding coefficient of friction (COF, μ) were automatically recorded. The experiment materials, such as virgin UHMWPE, surface porous GO/NaCl/UHMWPE composite, and 316 L stainless steel balls (diameter of 10 mm), were cleaned in acetone for 10 min each in an ultrasonic bath. The main component of synovial fluid is water. To control the variables of experimental factors, water was used as the lubricating medium in this experiment. The normal load is a significant parameter affecting the friction and wear properties of UHMWPE to metal. In this study, the friction and wear properties of porous polyethylene were investigated with load as variable. In the test progress, the UHMWPE and GO/NaCl/UHMWPE samples were fixed in the sample fixers with a 10 mL lubricant. The distilled water was used as the lubricant. The sliding velocity was 15 mm/s and the sliding duration was 11 min. The influence of the normal loads (20, 30, 50, 70, 90 N) on the tribological performance were investigated. All the tests were repeated three times, and the average values were calculated. The wear ratio (κ) was calculated with Equation (1):

(1) $$\kappa =\frac{v}{L\times F_{N}}$$

where *v* is the wear volume in mm^3^ measured with Keyence VK 9700 laser scanning microscopy [26,27]; *F*_N_ is the applied normal load in N; *L* is the total sliding distance in mm.

## 3. Results

### 3.1. Characterization of GO and Surface Porous GO/NaCl/UHMWPE Composites

#### 3.1.1. Characterization of GO

**Figure 3:**
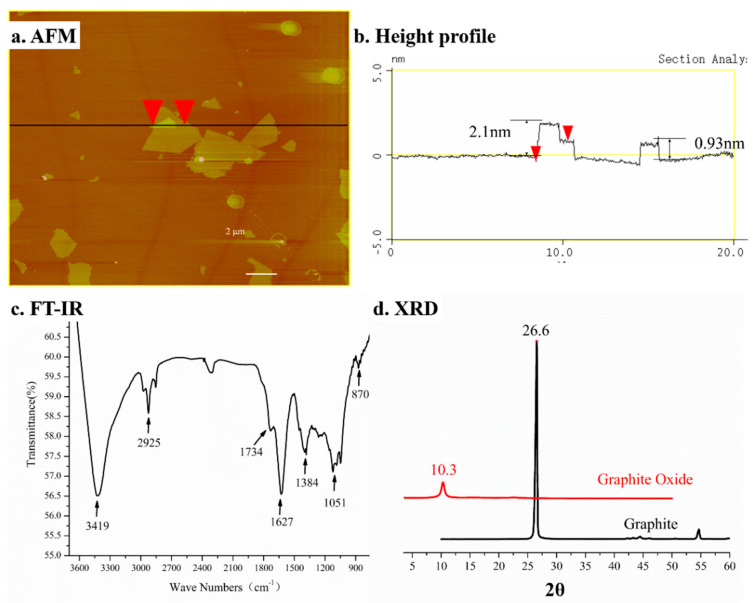
Characterization of GO. (**a**) AFM tapping mode image, (**b**) height profile, (**c**) FT-IR, and (**d**) XRD of GO.

The AFM images and height profiles of GO sheets are shown in Figure 3a,b. The thickness of the GO sheet was 0.93 nm and the stacking GO sheet was 2.1 nm. It is accordant with the values for single-layer GO sheet [28]. The chemical components of GO were evaluated by FT-IR, as shown in Figure 3c. The spectrum of O–H groups of GO at 3419 cm^−1^ was observed. The stretching vibrations of C=O and C=C of GO were 1734 cm^−1^ and 1627 cm^−1^, respectively. The vibration spectrum C–O in C–OH and C–O–C vibrations in epoxy were 1384 cm^−1^ and 1051 cm^−1^, respectively [29,30]. The interlayer spacing of graphite and GO was assessed by XRD according to the Bragg law shown in Equation (2):

(2) nλ=2dsinθ

The reflection of GO is a strong single peak at 2*θ* = 10.3°, illustrating that the GO layer spacing is larger as shown in Figure 3d. Due to oxide groups in GO, water molecules were trapped between the graphene oxide sheets [31,32]. No obvious peak is found in the profile of GO, indicating that graphite has been successfully oxidized to GO.

#### 3.1.2. Characterization of Surface Porous GO/NaCl/UHMWPE Composites

**Figure 4:**
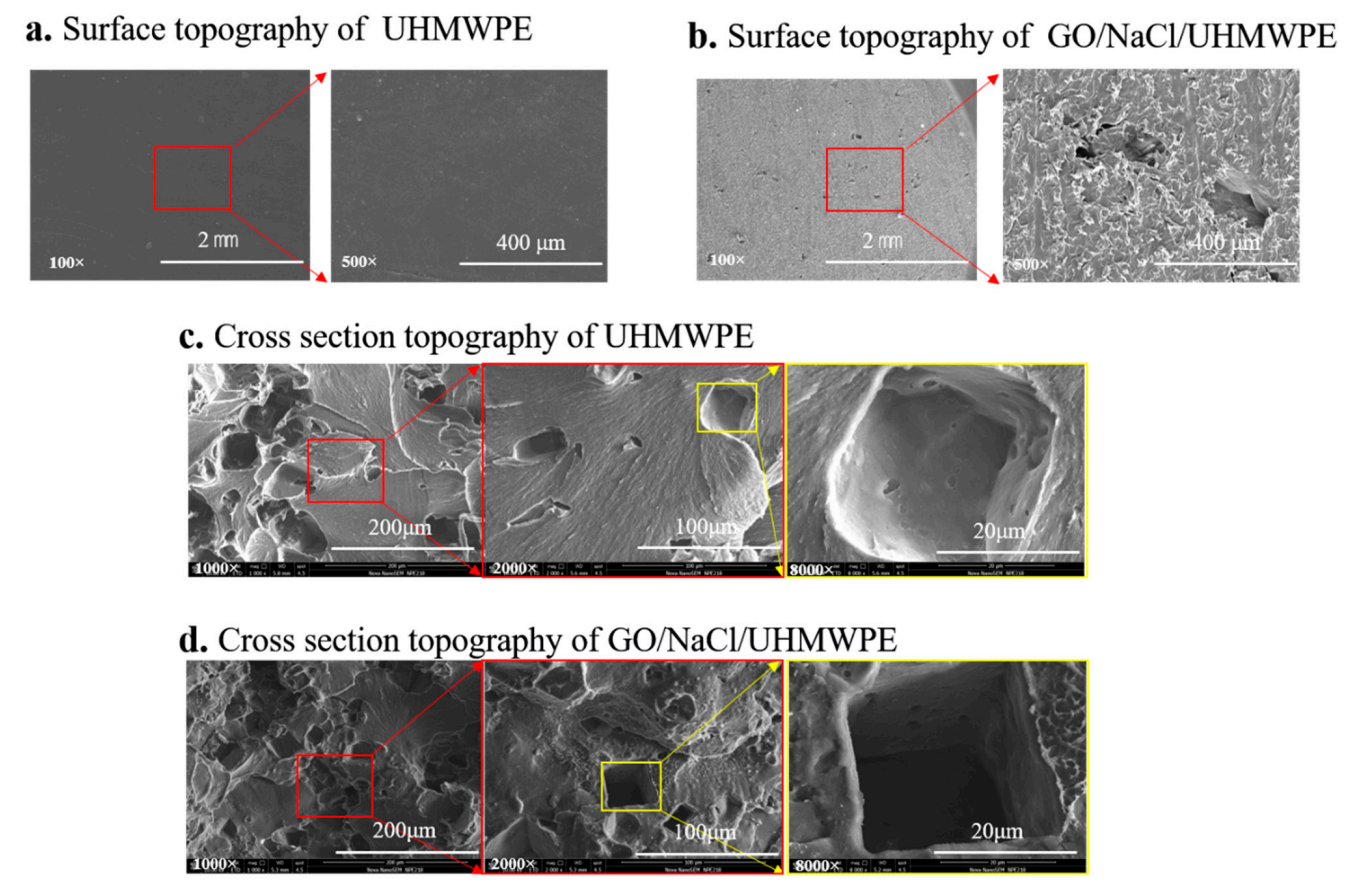
SEM of the surface and cross-section of UHMWPE and surface porous GO/UHMWPE composites.

Figure 4 shows the SEM of the surface and cross-section of UHMWPE and surface porous GO/NaCl/UHMWPE composites at different magnifications. From Figure 4b,c, it can be seen that more regular pores are present on the GO/NaCl/UHMWPE surface. Comparing with Figure 4b,c, it can be seen that the cross-section of UHMWPE material is relatively smoother with small pores, and the cross-section of surface porous GO/NaCl/UHMWPE composites was rough with the larger regular pores.

**Figure 5:**
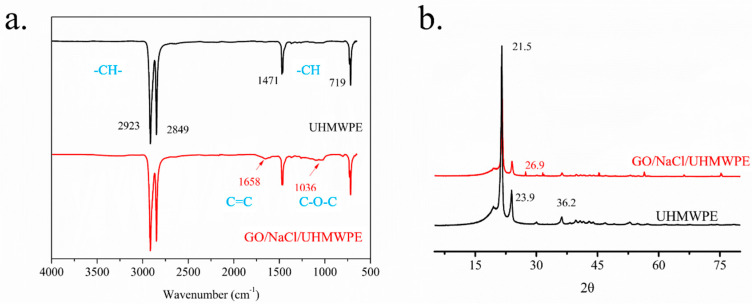
Characterization of the chemical structure of UHMWPE and surface porous GO/NaCl/UHMWPE composites with 1 wt.% GO and 10 wt.% NaCl. (**a**) FT-IR spectrum; (**b**) XRD spectrum.

FT-IR spectra were used to characterize the chemical structure of UHMWPE and surface porous GO/NaCl/UHMWPE composites with 1 wt.% GO and 10 wt.% NaCl, as shown in Figure 5a. The significant features with the asymmetric stretching vibration peaks (2923 cm^−1^) and symmetric stretching vibration peaks (2849 cm^−1^) of –CH– can be seen in the figure. The in-plane deformation vibration peaks of –CH appeared at 719 cm^−1^. Comparing with the peak strength of surface porous GO/NaCl/UHMWPE composites, it was found that the 1036 cm^−1^ and 1658 cm^−1^ peaks appeared in the strength for surface porous GO/NaCl/UHMWPE composites. This is because of the introduction of GO with oxygen-containing groups.

Figure 5b shows the XRD spectra of UHMWPE and surface porous GO/NaCl/UHMWPE composites with 1 wt.% GO and 10 wt.% NaCl. It can be seen that the XRD spectra of surface porous GO/NaCl/UHMWPE composites are similar to that of UHMWPE. The characteristic derivative peaks of UHMWPE occur at 21.5°, 23.9°, and 36.3°. The peak of surface porous GO/NaCl/UHMWPE composites occurs at 2*θ* = 26.9°. The surface porous structure would destroy the integrality of UHMWPE, and the X-ray value changed from 23.9° to 26.9° [31]. The results have shown that the surface porous GO/NaCl/UHMWPE composites did not degrade during the hot-pressing process. GO could be well dispersed in UHMWPE composites during the hot press processing. Therefore, GO has been successfully doped into UHMWPE matrix.

#### 3.1.3. Hardness of Surface Porous GO/NaCl/UHMWPE Composites

**Figure 6:**
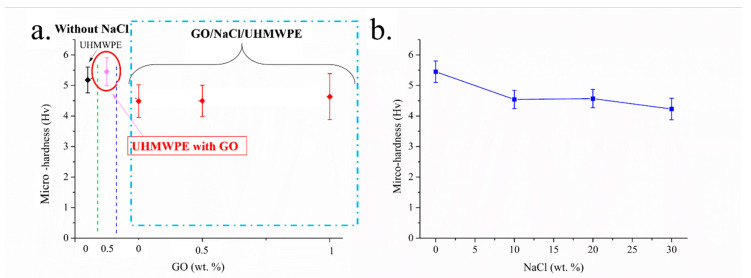
Influence of content of GO and NaCl on the UHMWPE hardness. (**a**) UHMWPE, UHMWPE with GO of 0.5 wt.%, UHMWPE with GO and NaCl (10 wt.%), (**b**) UHMWPE with GO of 1 wt.% with different content of NaCl.

The hardness curve of UHMWPE and surface porous GO/NaCl/UHMWPE were investigated. Figure 6a shows the influence of the content of GO on the hardness of GO/NaCl/UHMWPE with 10 wt.% NaCl. It can be seen that the hardness of UHMWPE was improved with the addition of GO of 0.5 wt.%. This is due to the GO nanoparticles’ small particle size, large surface curvature and excellent mechanical properties affecting the internal structure and hardness of polymer materials, effectively improving UHMWPE load-bearing capacity. Nevertheless, the hardness decreased with the addition of NaCl with 10 wt.%. Figure 6b shows the influence of the content of NaCl on the hardness of GO/NaCl/UHMWPE with 1 wt.% GO. It can be seen that the hardness of surface porous GO/NaCl/UHMWPE slightly decreased with the increase of NaCl content from 0 to 30 wt.%. This is due to the increased porosity of UHMWPE with NaCl dissolution. As shown in Figure 6, the hardness of porous UHMWPE increased with the increase of GO and decreased with the increased NaCl. To balance the UHMWPE surface pores and surface hardness, the GO/NaCl/UHMWPE composited with 1 wt.% GO and 10 wt.% NaCl.

#### 3.1.4. Contact Angle of Surface Porous GO/NaCl/UHMWPE Composites

**Figure 7:**
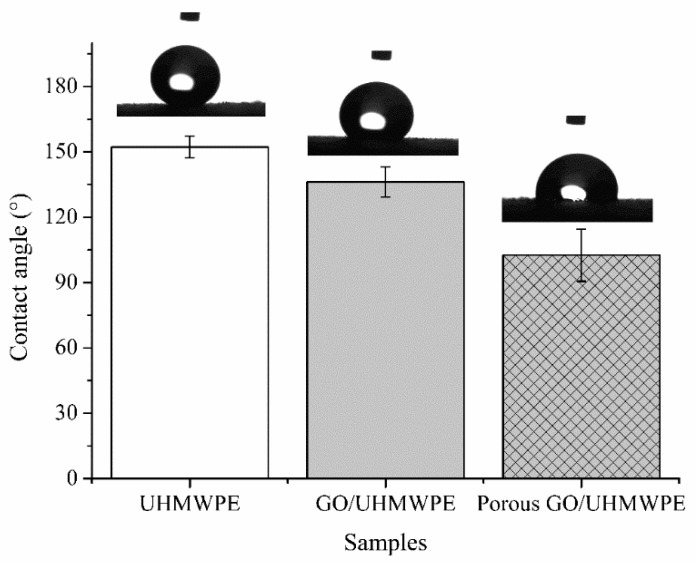
Contact angle of UHMWPE, GO/UHMWPE with GO 1 wt.% and surface porous GO/NaCl/UHMWPE composites with 1 wt.% GO and 10 wt.% NaCl.

Figure 7 shows the static contact angles of UHMWPE and surface porous GO/NaCl/UHMWPE composites with 1 wt.% GO and 10 wt.% NaCl. It can be seen that the static contact angle of UHMWPE material decreased with the addition of GO, and the static contact angle of surface porous GO/NaCl/UHMWPE composite further decreased with the addition of NaCl. This is due to the oxide functional groups on the GO surface and edge, which enhances the hydrophilic groups on the surface of the UHMWPE composite and reduces the static contact angle of the surface. The surface porosity of GO/NaCl/UHMWPE material was increased with the solution of NaCl. The static contact angle was further reduced. Hence, the surface wettability of GO/NaCl/UHMWPE was increased by adding GO and NaCl.

### 3.2. Tribological Performance of Surface Porous GO/NaCl/UHMWPE

The tribological performance of UHMWPE and surface porous GO/NaCl/UHMWPE composite and in particular the influences of GO, NaCl concentration, and lubrication on the coefficient of friction (COF) and wear rate (κ) were investigated.

#### 3.2.1. Influence of GO Content on Tribological Properties

**Figure 8:**
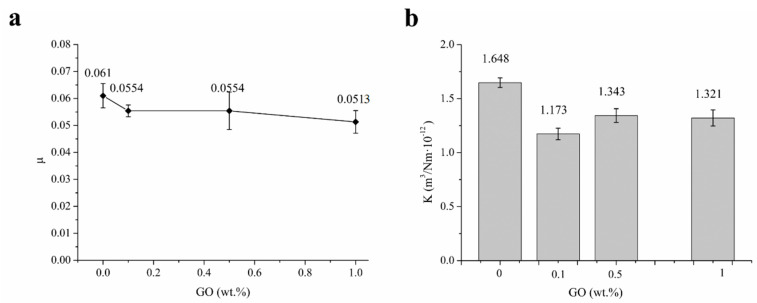
Effect of GO contents on tribological properties of UHMWPE and surface porous GO/NaCl/UHMWPE composites with 10 % NaCl in water, (**a**) the coefficient of friction (μ), (**b**) wear ratio (κ).

Figure 8 shows the effect of GO contents (0 to 1 wt.%) on the coefficient of friction (COF) and wear ratio (κ) of UHMWPE and surface porous GO/NaCl/UHMWPE composites with 10 % NaCl in water. From Figure 8a, it can be seen that COFs of surface porous GO/NaCl/UHMWPE composites decreased slightly compared with that of the porous UHMWPE without GO. With the increasing of GO contents from 0.1 wt.% to 1 wt.%, COF of surface porous GO/NaCl/UHMWPE composites slightly decreased from 0.056 ± 0.0046 to 0.051 ± 0.009. From Figure 8b, it can be seen that κ of surface porous GO/Nal/UHMWPE composites decreased slightly compared with that of the porous UHMWPE without GO. With the increase of GO content from 0.1 wt.% to 1 wt.%, κ of surface porous GO/NaCl/UHMWPE composites slightly increased from 1.18 ± 0.18 to 1.32 ± 0.23 m^3^/Nm·10^−12^. The addition of GO decreased the wear ratio because the GO reinforcement increased the UHMWPE elastic modulus. The layer-lattice structure of GO endows polymer with self-lubricating characteristics. These layers are linked by weak van der Waals bonds, which may be easily broken by sheer force under sliding conditions [33].

#### 3.2.2. Influence of NaCl Content on Tribological

**Figure 9:**
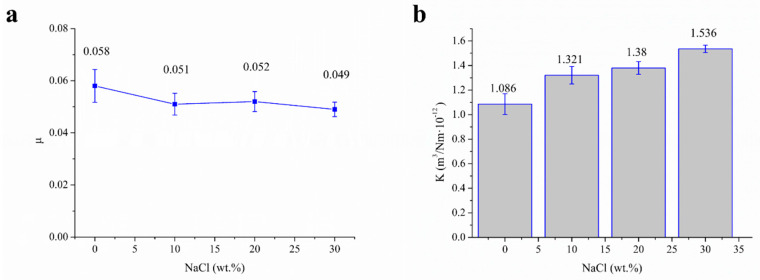
Effect of NaCl contents on tribological properties of UHMWPE and surface porous GO/NaCl/UHMWPE composites with 1% GO in water, (**a**) the coefficient of friction (μ), (**b**) wear ratio (κ).

Figure 9 shows the effect of NaCl contents (0, 10, 20, 30%) on COF and wear ratio (κ) of UHMWPE and surface porous GO/UHMWPE composites with 1% GO in the water. From Figure 9a, it can be seen that COFs of surface porous GO/NaCl/UHMWPE composites in water-based lubrication decreased gradually with the increase of NaCl contents from 5% to 30%. From Figure 9b, it can be seen that κ of surface porous GO/NaCl/UHMWPE composites in water-based lubrication increased gradually with the increasing of NaCl contents from 1.07 to 1.53 m^3^/Nm·10^−12^.

#### 3.2.3. Influence of Normal Loads on Tribological Performance

**Figure 10:**
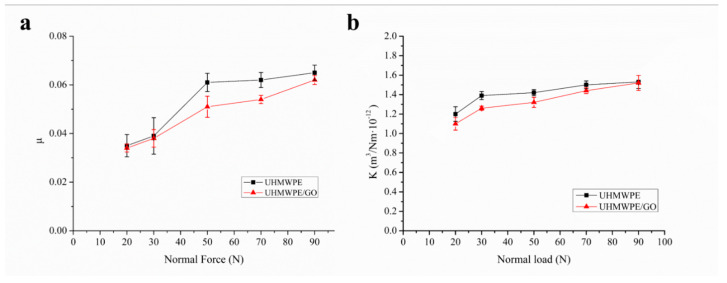
Effect of normal loads on the coefficient of friction and wear ratio of GO/UHMWPE composite material.

Figure 10 shows the effect of normal loads (20, 30, 50, 70, 90 N) on the COF and wear ratio (κ) of UHMWPE and surface porous GO/NaCl/UHMWPE composites with reciprocally moving at a speed of 30 mm/s in water. Figure 10a shows that COF of UHMWPE and surface porous GO/NaCl/UHMWPE composites increased gradually with the normal load increasing from 20 N to 90 N. When the load was greater than 50 N, COF presented a steady trend. Also, Figure 10b shows that the wear ratio gradually increased with the increase of normal load from 20 N to 90 N. Moreover, it can be seen from the figures that the COF and wear ratio of surface porous GO/NaCl/UHMWPE composite was lower than that of UHMWPE. This shows that the frictional and wear resistance of surface porous GO/NaCl/UHMWPE composites was better in water.

#### 3.2.4. Frictional and Wear Properties of Surface Porous UHMWPE

**Figure 11:**
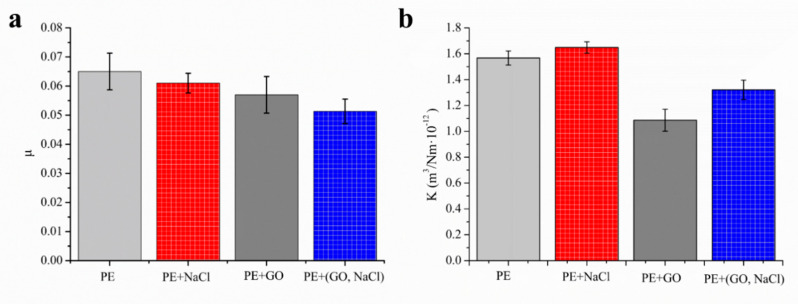
Coefficient of friction (**a**) and wear ratio (**b**) of UHMWPE, UHMWPE with NaCl, UHMWPE with GO, and UHMWPE with NaCl and GO.

Figure 11 shows the effect of the addition of GO (1 wt.%) and NaCl (10%) on COF and κ of four samples (UHMWPE, UHMWPE with NaCl, UHMWPE with GO, and UHMWPE with NaCl and GO) at the speed of 30 mm/s under normal load of 30 N in the water. From Figure 11a, it can be seen that the COF decreased with the addition of NaCl and GO. When GO and NaCl together presented in UHMWPE, the COF was less than the other samples. From Figure 11b, it can be seen that κ of UHMWPE with NaCl was higher than that of UHMWPE. The wear ratio of GO/NaCl/UHMWPE was less than the other samples. Also, the κ of UHMWPE with NaCl and GO was less than that of UHMWPE with GO and was larger than that of UHMWPE with NaCl.

## 4. Discussion

The pores structure of UHMWPE polymer which is similar to the porous articular cartilage was fabricated by NaCl and the UHMWPE mechanical properties were improved by GO. It is difficult for dry GO sheets to be evenly dispersed into the polymer [34]. In this research, the sedimentation method of GO in saturated NaCl was used to improve the GO sheets dispersion. When the GO and NaCl powder were mixed with UHMWPE with stirring, the GO sheets attached to UHMWPE powder and evenly dispersed in UHMWPE. The GO sheet and NaCl powder were dried with ethanol volatilization by continuous stirring. This prevented the GO sheet condensation. The chemical and mechanical characterizations have illustrated that the homogeneous NaCl and GO mixture was slowly dropped into ethanol solution. GO sheet was successfully synthesized by the characterization results of AFM, FT-IR, and XRD. The GO sheets were nano-size layers with oxygen chemical components.

The GO improved the hardness and wettability of UHMWPE. That was because the GO with NaCl increased the UHMWPE surface porous. In the friction process, the UHMWPE surface with pores would be destroyed, and the new UHMWPE surface presents new porous properties due to the solution of NaCl in the water. In this research, 1 wt.% GO and 10% NaCl filler were added to the UHMWPE material.

**Figure 12:**
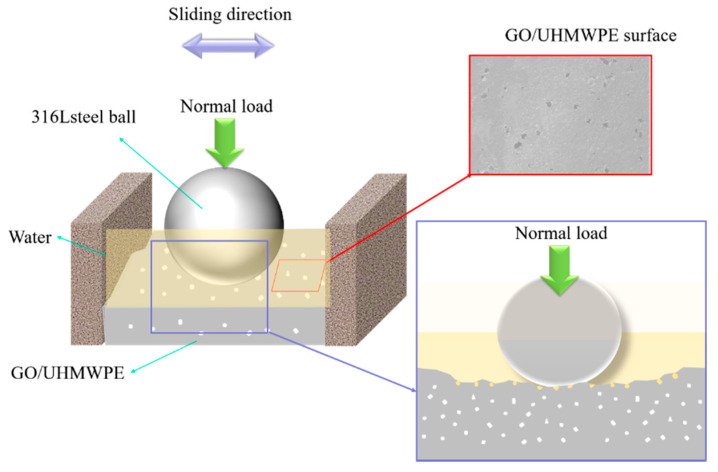
Schematic diagram of friction of porous GO/UHMWPE materials.

The frictional results reveal that surface porous GO/NaCl/UHMWPE presents the friction-reducing and anti-wear properties in water. It could be explained by the simplified schematic, shown in Figure 12. First, the adhesion part of the friction force *F_(f, adh)_* is determined by the shear strength τ of the materials at the interface and the real contact area *A_r_* between them [35]:

(3) $$F_{f,adh}=\tau \;A_{r}$$

The regular surface pores of GO/NaCl/UHMWPE are one crucial factor to improve tribological performance. The regular pores reduce the contact area between metal and UHMWPE.

Second, the porous structure improves the lubrication performance of lubricants by “encapsulating” [36]. Lubricants can be stored in the pores between the friction pairs. The shear strength was reduced with self-lubricating characteristics [37], thereby the COF and wear ratio were reduced. Besides, in the wear producer, the surface porous GO/NaCl/UHMWPE material is continuously worn, and new surface pores appear with the dissolution of NaCl. Moreover, the addition of GO sheet is composed of many laminated graphite planes, endowing it with self-lubricating characteristics [38], hence, improving the load-bearing capacity of the material.

## 5. Conclusions

In this research, GO/NaCl/UHMWPE composites were synthesized by hot pressing. The effects of GO and NaCl on the mechanical and tribological properties of surface porous GO-modified surface porous GO/NaCl/UHMWPE in water were investigated, which provided a theoretical basis for the preparation of composite artificial joint prosthesis materials.

(1) The sedimentation method of GO in saturated NaCl was used to improve the even dispersion of the GO sheets into the UHMWPE matrix. Considering the reinforcement of GO and destruction of NaCl, 1 wt.% GO and 10% NaCl filler were added to the UHMWPE material.

(2) The anti-friction and anti-wear properties of GO/NaCl/UHMWPE were improved with the water-based lubrication.

(3) In the friction process, with the dissolution of NaCl, new holes appear on the surface of GO/NaCl/UHMWPE, forming a virtuous circle of constant improvement of the anti-friction and anti-wear properties.

## Figures and Tables

**Table 1:** Mass ratios of surface porous GO/NaCl/UHMWPE composites.

Mass Ratios (wt.%)	1	2	3	4	5	6	7
UHMWPE	99	89.5	89	89.9	99	79	69
GO	0	0.5	1	0.1	1	1	1
NaCl	1	10	10	10	0	20	30

## Data Availability

The data presented in this study are available on request from the corresponding author.
